# McGrath® versus Macintosh laryngoscopes on hemodynamic response to intubation in elderly patients: a randomized clinical trial

**DOI:** 10.11604/pamj.2023.45.108.36562

**Published:** 2023-06-27

**Authors:** Salma Ketata, Mahdi Fourati, Rahma Derbel, Mariem Keskes, Karim Bouzid, Imen Zouche, Moncef Sallemi, Souha Kallel, Sami Fendri, Amine Zouari, Hichem Cheikhrouhou

**Affiliations:** 1 Department of Anesthesiology and Intensive Care Unit, Habib Bourguiba University Hospital, Sfax, Tunisia,; 2 Department of Oto-rhino-laryngology, Habib Bourguiba University Hospital, Sfax, Tunisia,; 3 Department of Visceral Surgery, Habib Bourguiba University Hospital, Sfax, Tunisia

**Keywords:** Orotracheal intubation, McGrath® videolaryngoscope, Macintosh laryngoscope, elderly patients

## Abstract

**Introduction:**

laryngoscopy and tracheal intubation induce catecholaminergic release. Our study aimed to evaluate the hemodynamic impact of orotracheal intubation by McGrath® compared to the Macintosh laryngoscope in the elderly.

**Methods:**

we conducted a prospective randomized clinical trial that included elderly patients proposed for a scheduled surgery under general anesthesia with orotracheal intubation and divided into 2 groups: patients who were intubated using the McGrath® (group V) and patients who were intubated using the Macintosh direct laryngoscope (group M). Heart rate (HR), systolic blood pressure (SBP), diastolic blood pressure (DBP), and mean arterial blood pressure (MAP), were recorded before induction of anesthesia (baseline), and at 1 min, 3 min, and 5 min after intubation. Our outcomes were the increase of SBP (∆ SBP), MAP (∆ MAP), and HR (∆ HR) between the two groups, during the 5 minutes following the start of the orotracheal intubation, intubation time and the incidence of its related complications.

**Results:**

sixty patients were included and randomized into 2 groups of 30. The average age of our sample was 70±6 years with a sex ratio of 1.22. Most of the patients were operated on for orthopedic, urologic, or abdominal surgery. There were no statistically significant differences between the two groups in terms of demographic characteristics and the duration of anesthesia (p> 0.05). The intubation time was significantly increased in group M (p≤0.001). There was a significant difference in SBP, MAP, and HR values at 1 min after orotracheal intubation compared with the baseline values in Group V(P<0,05) and Group M (p < 0.05). There was a significant increase in the first minute after tracheal intubation in terms of SBP (151±42 vs 134.5±26 mmHg, p=0.012), MAP (114±4 vs 102±17 mmHg, p=0.015), DBP (89±32 vs 84±16 mmHg, p=0.01), and HR (99.5±10 vs 94.5±2 b/min, p=0.008) when group M was compared to group V. The ∆SBP was significantly different between group M (∆SBP = 36.2±23.5mmHg) and group V (∆SBP= 30.77±21.6mmHg) (p = 0.005). There were 4 ventricular arrhythmias in group M versus zero in group V (p <0.0001). The postoperative sore throat was significantly decreased in group M vs V (p=0.036).

**Conclusion:**

the McGrath® videolaryngoscope decreased the hemodynamic fluctuations due to endotracheal intubation in elderly patients.

## Introduction

Direct laryngoscopy with the Macintosh laryngoscope is commonly used in orotracheal intubation. Laryngoscopy and intubation of the trachea could induce hemodynamic variations like hypertension and tachycardia [1], which can be the cause of stroke, myocardial infarction, and cardiac arrhythmias in the perioperative period [2,3]. The mechanical stimulation of the upper airways caused by laryngoscopy and tracheal intubation activates the sympathetic system, which leads to cardiovascular [4,5].

Alongside direct laryngoscopy, indirect laryngoscopy is booming since the beginning of the 21^st^ century. Several devices are available such as the Airtraq® (Prodol Meditec S.A., Vizcaya, Spain), the Glidescope® (GSD, Verathon, Bothell, WA, USA), and the McGrath® (Medtronic, USA). The McGrath® videolaryngoscope is a device with a rigid blade. It is designed to facilitate tracheal intubation in patients with normal or difficult airways [6]. This study aimed to evaluate the hemodynamic impact of orotracheal intubation by McGrath® compared to the Macintosh laryngoscope in the elderly patients proposed for scheduled surgery.

## Methods

**Study design and setting:** we conducted a prospective randomized controlled clinical trial in the anesthesia intensive care unit, including elderly patients proposed for a scheduled surgery under general anesthesia with orotracheal intubation.

**Study population:** this study included patients aged over 65 years with American Society of Anesthesiologists score (ASA) I and II, and candidates for elective surgery, greater than 30 minutes and less than 120 minutes, under general anesthesia with orotracheal intubation. Non-inclusion criteria were patient refusal, surgical emergency, predicted difficult airway management, preoperative hemodynamic instability, beta-blockers intake, unstable heart disease, inherited cardiac arrhythmias, and intracranial hypertension (IH). Difficult or traumatic Airway management was excluded from the study. The calculation of the number of subjects required was carried out for a minimum difference of 20 mmHg and a standard deviation of 20 mmHg for the ∆PAS (∆SBP=SBPmax - SBP0) to be demonstrated, either in the bilateral hypothesis, with type I and II risks (alpha and beta) to 5%. We determined a minimum sample size of 26 subjects per group to ensure a study power of 95%. Thus, we decided to include 78 patients to compensate for possible attrition.

**Randomization and allocation:** the randomization was made using a web-based randomization sequence. The patients were then divided into two groups: patients who were intubated using the McGrath® videolaryngoscope (group V) and patients who were intubated using the Macintosh direct laryngoscope (group M).

**Interventions:** a first anesthesiologist had provided, at least 48 hours before the surgery, the pre-anesthetic consultation. He carried out a rigorous clinical examination, an adjustment of the medications, and checked the inclusion and exclusion criteria. In the operating room, noninvasive blood pressure, ECG, and pulse oximeter (SpO_2_) monitoring were performed. 3 ml/kg of crystalloids was administrated before induction of anesthesia. After 3 min of preoxygenation with 100% oxygen, general anesthesia was induced with 3 mg/kg of propofol and 1 mg/kg of succinylcholine. The laryngoscopy was performed by an experienced anesthesiologist (more than 50 laryngoscopies in each technique) different from the one who carried out the randomization of the patients. Orotracheal intubation was performed using endotracheal tubes in sizes 7.5 mm and 7 mm, for men and women, respectively. Intubation time was recorded as the time from the insertion of the laryngoscope blade into the mouth to the inflation of the airway cuff. The intratracheal position of the tube and the symmetry were checked by pulmonary auscultation and capnography. The assessment of the hemodynamic response (systolic blood pressure (SBP), mean arterial blood pressure (MAP), diastolic blood pressure (DBP), and heart rate (HR)) was conducted during the 5 minutes following the start of the orotracheal intubation (before induction of anesthesia (baseline)=T0, and at 1 min=T1, 3 min=T2, and 5 min=T3 after intubation. During this period, no painful stimulation was performed. Anesthesia was maintained with Remifentanil (0.25 μg/kg/min), Cisatracurium (0.15 mg/kg then 0.03 mg/kg every 20 minutes), and Sevoflurane with a target minimum alveolar concentration of 1%. The onset of major cardiac events was noted. In the post-anesthesia care unit (PACU), an anesthesiologist different from the one who performed the intubation carried out an assessment of laryngeal morbidity, in particular hoarseness and sore throat, two hours after tracheal extubation.

**Data collection:** gender, age, height, body mass index (BMI), and ASA score were noted. Systolic blood pressure (SBP), mean arterial blood pressure (MAP), diastolic blood pressure (DBP), and heart rate (HR)) were collected during the 5 minutes following the start of the orotracheal intubation (before induction of anesthesia (baseline)= T0, and at 1 min=T1, 3 min=T2, and 5 min=T3 after intubation. The onset of major cardiac events, the intubation time, and postoperative laryngeal morbidity were also collected.

**Variables:** our primary outcome was the increase in systolic blood pressure (SBP) defined as a difference in SBP (∆SBP=SBPmax - SBP0), between the two groups, during the 5 minutes following the start of the orotracheal intubation. The secondary outcomes were the increase of mean arterial pressure (MAP) or heart rate (HR) after laryngoscopy defined as a difference of MAP (∆MAP=MAPmax - MAP0) equal to or greater than 20 mmHg or a difference of HR (∆HR=HRmax - HR0) equal or greater than 20 bpm, between the two groups, during the 5 minutes following the start of the orotracheal intubation and the incidence of complications related to intubation such as unpredictable difficult intubation, heart rhythm disorders, and postoperative sore throat

**Statistical analysis:** Statistical Package for the Social Sciences (SPSS) version 25.0 software was used for data entry and analysis. The description of the qualitative variables was carried out by calculating the percentages. For the quantitative variables, we checked the normality of the distribution by the Shapiro-Wilk test. Then we estimated the means with the standard deviations (SD) or medians with the semi-interquartile ranges (SIR) depending on the distribution. For the comparisons of qualitative variables between the two groups, we used the Chi-square test of the Pearson or Fisher test when needed. As for the comparison of the quantitative variables, we used the t-student test for the comparison of means in the case of normal distribution and the Mann-Whitney test in the case of non-Gaussian distribution. The comparison of hemodynamic data including repeated measures within groups was performed using repeated measures analysis of variance (ANOVA test), where the calculated p-value exceeded the critical value for the 0.05 probability level. A linear correlation analysis (Pearson test) and an analysis of covariance between ∆SBP and SBP (baseline), are carried out to study the existing dependence between these two variables as well as the possible interaction of the technique on this dependence. A p-value less than 0.05 was considered significant.

**Ethical considerations:** this study was conducted after approval of the southern protection committee of People (C.P.P.SUD) under the aegis of the Health Ministry of the Tunisian Republic reference CPP SUD N°0274/2020 and written informed consent of patients.

## Results

**Figure 1:**
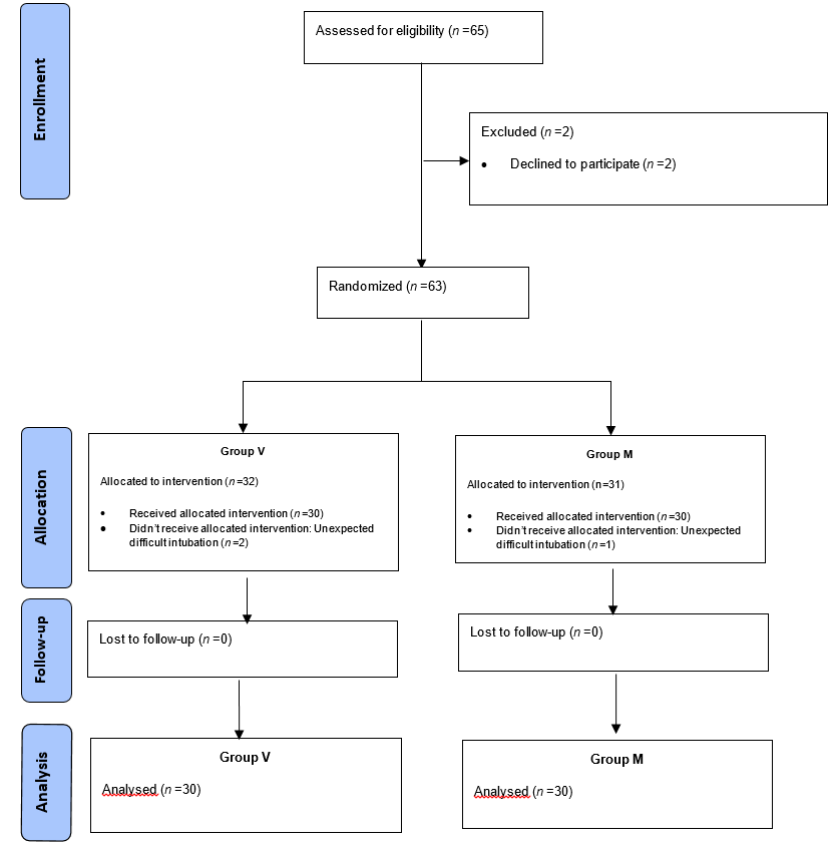
flow chart showing videolaryngoscope (group V) and Macintosh group (group M)

Sixty-five patients were included and 5 patients were excluded. We analyzed 60 patients divided into two groups: 30 in group M, and 30 in group V (Figure 1).

**Baseline characteristics:** the average age of our sample was 70±6 years. It was made up of 27 men and 33 women with a sex ratio of 1.22. Most of the patients were operated on for orthopedic, urologic, or abdominal surgery. There were no statistically significant differences between the two groups in terms of demographic characteristics and the duration of anesthesia (P > 0.05) (Table 1). Mallampati scores were similar in both groups. There was a statistically significant difference between Group V (20±7.6 s) and group M (14.4±5.4 s) in terms of intubation time (P ≤ 0.001) (Table 1).

**Table 1:** comparison of demographic data and intubation characteristics between the 2 groups

	Group V (n=30)	Group M (n=30)	P-value
**Age**	70.97 ± 6.21	70.67 ± 5.91	0.87*
**Gender (F/M)**	16/14	17/13	0.795†
**BMI (Kg/m^2^)**	23.05 ± 2.63	23.82 ± 3.01	0.471*
**ASA (I/II)**	19/11	13/17	0.606†
**Mallampati score (I/II/III/IV)**	8/16/6/0	4/16/9/1	0,71†
**Time for intubation (sec) ± SIR**	20 ± 7.6	14.4 ± 5.4	≤0.001˟

Group V: Videolaryngoscope, Group M: Macintosh group, n=number, BMI: body mass index, F: female, M: male, ASA: American Society of Anaesthesiologists score, sec: seconds, SIR: Semi interquartile range, (*): Student's t test, (†): Chi-square test, (˟): Mann-Whitney test

### Primary and secondary outcomes

**Hemodynamic outcomes:** there was a significant difference in SBP, MAP, and HR values at 1 min after orotracheal intubation compared with the baseline values in Group V (P < 0.05) (Table 2). In Group M, there was a significant difference in SBP, MAP, and HR values in the first minute after intubation compared with the baseline values (Table 2). There was a significant increase in the first minute after tracheal intubation in terms of SBP (151±42 vs 134.5±26 mmHg, p=0.012), MAP (114±4 vs 102±17 mmHg, p=0.015), DBP (89±32 vs 84±16 mmHg, p=0.01), and HR ( 99,5±10 vs 94,5±2 b/min, p=0,008) when group M was compared to group V (Table 2). The ∆SBP was significantly different between group M (∆SBP = 36.2 ± 23.5) and group V (∆SBP= 30.77 ± 21.6) (p = 0.005). A linear correlation study and an analysis of covariance (Pearson test) made it possible to study the relationship between the dependent variable (∆SBP) and its covariate (SBP (baseline)) depending on the technique used. These two variables had a non-significant correlation (correlation coefficient r = 0.04, p = 0.974). The hemodynamic response was different between the two techniques independently of baseline values.

**Table 2:** comparison of hemodynamic changes between the two groups

Measures	Group V (n=30)	Group M (n=30)	P-value
**HR (beats/min) ± SIR**			
Baseline	80 ± 14	73 ± 15	0.051
After intubation			
1 min	94.5 ± 20#	99.5 ± 10#	0.008×
3 min	78 ± 14	80.5 ± 9	0.609
5 min	72 ± 16	72 ± 18	0.662
**SBP (mmHg) ± SIR**			
Baseline	119 ± 15	120.5 ± 16	0.953
After intubation			
1 min	134.5 ± 26#	151 ± 42#	0.012×
3 min	104 ± 27	113 ± 21	0.052
5 min	97 ± 21	105.5 ± 25	0.166
**DBP (mmHg) ± SIR**			
Baseline	71.5 ± 14	71 ± 15	0.117
After intubation			
1 min	84 ± 16	89 ± 32	0.01×
3 min	61 ± 47	66 ± 50	0.424
5 min	59 ± 42	58.5 ± 61	0.301
**MAP (mmHg) ± SIR**			
Baseline	87 ± 15	90 ± 11	0.125
After intubation			
1 min	102.5 ± 17#	114 ± 40#	0.015×
3 min	75 ± 13	82 ± 17	0.066
5 min	72.5 ± 8	76 ± 10	0.303


Group V= Videolaryngoscope group, Group M= Macintosh group; HR: heart rate; SBP: systolic blood pressure; DBP: diastolic blood pressure; MAP: mean arterial pressure; min: minutes, n=number, data are presented as medians ± SIR: Semi interquartile range; (#)= P < 0.05 compared with baseline values after significant repeated measures ANOVA; (×) = P < 0.05 between groups based on the Mann Whitney test.

**Intubation related complications:** six tracheal intubations required an Eschmann stylet or external laryngeal pressure in group M versus 2 in group V, with no significant difference between the two groups (p=0.129). There were 4 ventricular arrhythmias in the L group versus zero in group V (p <0.0001). No asystole and no cardiac repolarization abnormities were recorded. Hoarseness and sore throat were observed in 20 patients (66.6%) in group M and 10 patients (33.3%) in group V. There was a significant difference in terms of postoperative laryngeal morbidity between the groups (p=0.036).

## Discussion

We conducted a prospective randomized clinical trial to evaluate the hemodynamic impact of orotracheal intubation by McGrath® compared to the Macintosh laryngoscope in the elderly patients proposed for a scheduled surgery under general anesthesia with orotracheal intubation. We included 60 patients and divided them into 2 equivalent groups: patients who were intubated using the McGrath® (group V) and patients who were intubated using the Macintosh direct laryngoscope (group M). This study showed that the McGrath® videolaryngoscope decreased hemodynamic fluctuations and complications due to endotracheal intubation in elderly patients with fewer heart complications or postoperative laryngeal morbidities.

Over the last few decades, several studies listed in the anesthesia literature have addressed the hemodynamic impact of laryngoscopy and orotracheal intubation. In 1951, King *et al*. [7] demonstrated that laryngoscopy comes with significant hemodynamic variations such as tachycardia, arterial hypertension, and arrhythmias. However, the mechanisms of these variations have not been eluded. In a prospective study, Knight *et al*. [8] studied the hemodynamic impact of three laryngoscopy techniques (Macintosh curved blade, Miller straight blade, and lighted stylet) and found that the intensity of the hemodynamic response is correlated with the lifting forces applied to expose the glottis and the duration of laryngoscopy. Siddiqui *et al*. [9] showed that the force applied during laryngoscopy, the duration of laryngoscopy, and the number of attempts could aggravate the sympathetic response during laryngoscopy. The force exerted on the tongue during this procedure could be around 30-50 Newton [3-5]. As the alignment of the oropharyngeal axis is not required to visualize the glottis with the McGrath®, less force is required during intubation with this device [10]. In fact, with the McGrath videolaryngoscope, oral, pharyngeal, and laryngeal anatomical structures do not need to be aligned for the airway anatomy and vocal cords to be seen clearly, which means less lifting force is required to expose the glottis. Kato and al and Wallace *et al*. [11,12] noted on a mannequin that the force exerted on contact with the maxillary incisors was 3 times less with the McGrath® than with the Macintosh laryngoscope (141.1±15.7 kg for Macintosh compared with 48.7±6.7 kg for McGrath®, p<0.05), which suggests a lower risk of fracture or dental dislocation during tracheal intubation with the McGrath®. Hoshijima *et al*. [13] in a meta-analysis found less bleeding with the McGrath® than with the Macintosh blade.

In our study, the intubation time obtained with the McGrath® was greater than that obtained with the Macintosh laryngoscope. In a study of 120 patients, Kaur *et al*. [14] showed that the McGrath® significantly shortened the duration of intubation compared to the Macintosh laryngoscope, which would attenuate the hemodynamic impact of tracheal intubation. In contrast, Walker *et al*. [15] noted that a first-year anesthesia student took 17 seconds longer during intubation with the McGrath® than with the Macintosh laryngoscope. The time required to remove the intubation style used in McGrath® could explain this difference.

Mechanical stimulation of pharyngeal and laryngeal proprioceptors during laryngoscopy and tracheal intubation induces the secretion of cortisol and catecholamines, and therefore a subsequent increase in HR (by about 20%), blood pressure (BP) (40-50%), pulmonary capillary pressure (Pcap) and intracranial pressure (ICP) [4,8,16]. The resulting increase in myocardial consumption puts the body at risk of myocardial infarction, arrhythmias, heart failure, and acute lung edema. In the elderly, the hemodynamic impact of tracheal intubation is more pronounced than in the general population; in fact, the main characteristic of the cardiovascular system in the elderly is its difficulty in adapting to stressful situations [17,18]. This is due to a higher prevalence of cardiovascular comorbidities and the atypical aspect of these. These pharmacokinetic, pharmacodynamic, and autonomic changes accentuate the hemodynamic response to tracheal intubation [19,20]. In our study, there was a significant increase in the first minute after orotracheal intubation in terms of SBP, MAP, DBP, and HR with group M compared to group V. In a prospective randomized double-blind study including 96 elderly patients, Colak *et al*. [20] showed that compared to the Macintosh laryngoscope, the McGrath® videolaryngoscope induced fewer significant hemodynamic changes, including HR, SBP, and MAP, up to 1 minute after orotracheal intubation.

We noted a tendency for arrhythmia (ventricular extrasystoles) in group M when compared to group V. This may be due to a greater adrenergic discharge caused by Macintosh laryngoscopy, related to the intensity of the force applied [9]. No repolarization disorders were noted, suggesting that no significant coronary events occurred in either group. The laryngoscopy technique influences the postoperative laryngeal morbidity represented by a sore throat and hoarseness as shown by Kriege *et al*. [21] in a multicentric randomized trial entitled EMMA. In our study, postoperative laryngeal morbidity represented by a sore throat and hoarseness were lower in group V. This is consistent with the hemodynamic changes noted, supporting the nociceptive origin of these side effects. Our study has some limitations. An analysis of these immediate hemodynamic variations via invasive arterial monitoring remains ideal. However, invasive arterial monitoring for ASA I and II patients without coronary artery disease is not recommended in our daily practice.

## Conclusion

In elderly patients, the McGrath videolaryngoscope, in comparison to the Macintosh direct laryngoscope, was proven to decrease the hemodynamic alterations that are due to tracheal intubation with less postoperative laryngeal morbidity. However, the duration of the intubation is significantly shorter with the Macintosh direct laryngoscope.

### What is known about this topic

- *Laryngoscopy causes significant hemodynamic variations such as tachycardia, arterial hypertension, and arrhythmias*;
- *In the elderly, the hemodynamic impact of tracheal intubation is more pronounced than in the general population*;
- *The McGrath® videolaryngoscope is a device with a rigid blade; it is designed to facilitate tracheal intubation in patients with normal or difficult airways*.

### What this study adds

- *The McGrath® videolaryngoscope decreased the hemodynamic fluctuation due to endotracheal intubation in elderly patients*;
- *The McGrath® videolaryngoscope decreased several serious complications due to endotracheal intubation in elderly patients*;
- *The McGrath® videolaryngoscope decreased postoperative laryngeal morbidities due to endotracheal intubation in elderly patients*.
